# Supplementary material for: Impact of Multiple Factors on the Incidence of Developmental Dysplasia of the Hip: Risk Assessment Tool
Source: Medicina (Kaunas). 2022 Aug 25;58(9):1158. doi: 10.3390/medicina58091158 (PMC9502116; doi:10.3390/medicina58091158)
Supplement: Supplementary file 1 [file medicina-58-01158-s001.zip › Supplementary File S2.pdf]

Table S1. Potential risk factors influencing the occurrence of DDH. Statistical significance assessed by the Chi2 test with appropriate corrections.

**Variable**	**DDH**		χ^2
	**Yes**	**No**	
**Multiple pregnancy**			
Yes	2 (0.06%)	92 (2.97%)	0.392
No	136 (4.38%)	2872 (92.59%)	
**Macrosomia > 4000 g**			
Yes	17 (0.55%)	302 (9.74%)	0.420
No	121 (3.90%)	2662 (85.82%)	
**Transverse position**			
Yes	0 (0.00%)	1 (0.03%)	1.00
No	138 (4.45%)	2963 (95.52%)	
**Delivery ≥ 42 week**			
Yes	6 (0.19%)	59 (1.90%)	0.112
No	132 (4.26%)	2905 (93.65%)	
**First childbirth**			
Yes	75 (2.42%)	1692 (54.55%)	0.525
No	63 (2.03%)	1272 (41.015)	
**APGAR Score < 10**			
Yes	16 (0.52%)	352 (11.35%)	0.920
No	122 (3.93%)	2612 (84.20%)	

χ^2 - the significance of p for χ^2
